# Intervention to promote preventive dental care for older Korean-American Medi-Cal enrollees in Los Angeles

**DOI:** 10.1186/s12903-024-04113-z

**Published:** 2024-03-14

**Authors:** Yuri Jang, Juyoung Park, Chaeyoon Park, Shinyi Wu, Piedad Suarez-Durall, Soondool Chung, Miyong T. Kim

**Affiliations:** 1 Edward R. Roybal Institute On Aging, Suzanne Dworak-Peck School of Social Work, University of Southern California, 669 West 34th Street, Los Angeles, CA 90089-0411 USA; 2 Department of Social Welfare, Ewha Womans University, Seoul, Republic of Korea; 3 Department of Social Welfare, Soongsil University, Seoul, Republic of Korea; 4 Viterbi School of Engineering, University of Southern California, Los Angeles, USA; 5 Herman Ostrow School of Dentistry, University of Southern California, Los Angeles, USA; 6 School of Nursing, University of Texas at Austin, Austin, USA

**Keywords:** Oral health education, Digital intervention, Preventive dental care, Older immigrants, Korean-Americans, Limited English proficiency

## Abstract

**Background:**

In California, preventive dental care is covered by Medi-Cal (California’s Medicaid program). However, many beneficiaries do not use their dental benefits. Given that a lack of knowledge about oral health and insurance coverage contributes to this underutilization, promoting the use of dental benefits among eligible individuals via an educational program is imperative. Responding to the particular needs of older immigrants with limited English proficiency, we developed a digital oral health intervention for older Korean-American Medi-Cal enrollees in Los Angeles. This educational intervention is designed to be delivered via computers and the Internet. It consists of a 15-min self-running PowerPoint presentation narrated in Korean with links to additional information on the Internet. The slides contain information about the basic etiology of oral diseases, oral hygiene, common myths about oral health and dental care, Medi-Cal coverage of preventive dental care, and how to find a dental clinic.

**Methods:**

We pilot tested the intervention with 12 participants to examine its feasibility and acceptability. We also obtained participants’ qualitative feedback about the intervention.

**Results:**

A post-intervention quantitative assessment yielded high participant satisfaction and improved oral health and dental care knowledge. Participant responses to the intervention yielded four themes: (1) content and structure, (2) linguistic and cultural aspects, (3) delivery mode, and (4) additional concerns and suggestions.

**Conclusions:**

Our findings confirm the intervention’s feasibility and acceptability and suggest further refinement.

## Background

The importance of oral health for overall health and well-being is widely known [1–4]. Systematic reviews have demonstrated the benefits of preventive dental care for early detection and better control of oral diseases [5, 6]. Although oral health is a national public health priority [1, 4], many segments of the U.S. population experience a disproportionate burden of oral disease and inequities in dental care [2, 3]. Among those at high risk are older immigrant populations with language barriers [7].

Limited English proficiency (LEP) is a term used to describe individuals who do not speak English as their primary language and have limited ability to read, speak, write, or understand English [8]. According to the 2010 Census, more than 18% of the U.S. population (47 million people) do not speak English as their primary language, and more than 25 million speak English less than *very well* [8, 9]. The oral health burdens of LEP populations are particularly high. Older adults with LEP are significantly worse off (at a risk 1.68–3.47 times higher) than their English-speaking counterparts on measures of oral health and dental care [10–12]. Despite federal and nonfederal initiatives to address the healthcare challenges of language minorities [13, 14], disparities persist in preventive dental care even among those who have health insurance.

Although preventive dental care in California is covered by Medi-Cal (California’s Medicaid program) [15], many beneficiaries do not use their dental benefits. In a study [16] using data from the 2017 California Health Interview Study (CHIS), despite having dental coverage, more than 40% of Medi-Cal beneficiaries had not visited a dentist in the past year. The underutilization rate is even higher among older immigrants with LEP [10–12, 17]. The lack of knowledge about oral health and insurance coverage contributes to this underutilization [7, 10–12]; thus, developing targeted oral health education to promote preventive dental care is imperative.

The target group in this study was older Korean-American Medi-Cal enrollees in Los Angeles. Korean Americans present the fifth-largest Asian-American subgroup, with a notably high LEP rate [8]. Approximately one-third of all Korean immigrants in the U.S. reside in California, and over two-thirds of California’s Korean population is concentrated in greater Los Angeles [9]. Due to their relatively recent immigration to the U.S., most older Korean Americans are foreign-born and face cultural and linguistic barriers in the host society [10]. Older Korean Americans with LEP are particularly vulnerable in the areas of health and healthcare [18, 19]. In a qualitative study with a small sample of older Korean Americans in Los Angeles [20], we found unmet oral health needs, unawareness of preventive oral healthcare, and underutilization of dental services, underscoring the urgency of targeted oral health education.

This study describes a digital oral health education intervention program for older Korean-American Medi-Cal enrollees in Los Angeles designed to promote preventive dental care. We tested the intervention for feasibility and acceptability using a small sample of our target population. Pilot testing was necessary because the digital delivery of education is new to older immigrants [21]. Furthermore, ensuring an intervention’s linguistic and cultural appropriateness during development is vital [22]. Indeed, pilot testing is highly recommended in the National Institutes of Health stage model of intervention development [23]. Here, as part of the intervention development, we present the initial findings on the intervention’s feasibility and acceptability. Qualitative feedback from participants on technical and cultural appropriateness will inform future intervention development and implementation.

## Methods

### Development

The intervention consisted of 15 min of oral health education delivered in the Korean language via computers and the Internet. We chose educational content from an extensive literature review and relevant public educational programs in the U.S. and South Korea (e.g., information from the Centers for Disease Control and Prevention, the American Dental Association, and the Korea Health Promotion Institute). Topics included the basic etiology of oral diseases, oral hygiene, common myths about oral health and dental care, Medi-Cal coverage of preventive dental care, and how to find a dental clinic. This content was formatted as a PowerPoint presentation using an automated self-running series of 42 slides presented and narrated in Korean. The presentation also included a link to a YouTube presentation on using dental floss from the Korean Health Promotion Institute (https://www.youtube.com/watch?v=udDOCZqXMgs) and a link to the Medi-Cal Dental Program’s directory of dental care providers (https://ko.smilecalifornia.org). With the entry of one’s zip code and preferred language, the latter website generates a list of nearby dental clinics with language concordance that accept Medi-Cal. A tutorial on this search step is part of the intervention, which was reviewed by content and cultural experts.

### Recruitment, pilot testing, and evaluation

The eligibility criteria for the pilot testing were (1) self-identified Korean American, (2) aged 65 years or older, (3) living in Los Angeles, (4) enrolled in Medi-Cal, and (5) no preventive dental care in the past year. We used a convenience sample, and 12 participants were recruited from a senior center in Los Angeles’ Koreatown neighborhood. The center is a nonprofit social service agency operated by bilingual staff to meet older Korean Americans’ educational and recreational needs. The intervention was offered in private rooms at the center equipped with a computer and the Internet. Trained research personnel set up the PowerPoint presentation on the computer, assisted with operating the equipment, and conducted the assessment. The Institutional Review Board of the University of Southern California approved the project.

Before the presentation of the educational intervention, the participants completed a brief survey of sociodemographic characteristics, oral health needs, awareness of Medi-Cal’s coverage of preventive dental services, prior experience with oral health education, and comfort level with the use of computers and the Internet. A post-assessment survey was also conducted. Five items from the Client Satisfaction Questionnaire [24] were used to measure the level of satisfaction with the educational program. Participants rated the intervention’s quality, the extent to which it met their needs, and their willingness to recommend it to others. Each response was coded on a 4-point response format. Total scores ranged from 5 to 20, with higher scores indicating greater satisfaction with the program. The scale’s internal consistency in this sample was high (α = 0.93). Participants were also asked about perceived changes in knowledge about oral health, willingness to use preventive dental care, and self-sufficiency in dental care navigation. A qualitative interview was also conducted to obtain the participants’ feedback. The interview ranged from 30 to 45 min. Participants were encouraged to comment on any aspects of the intervention, but when necessary, probes were used to solicit input on technical and cultural elements.

## Results

### Descriptive characteristics of the sample

Twelve older Korean Americans participated aged 65 to 89 years (*M* = 73.7, *SD* = 8.17). Over 83% were women, approximately 67% were married, and over 90% had at least a high school education. Their years in the U.S. ranged from 10 to 45, with an average of 27.9 (*SD* = 11.3) years. All participants had LEP, meaning all spoke English less than *very well*. Most (83.3%) rated their oral health as *fair* or *poor*. More than half (58.3%) of the respondents did not know that Medi-Cal provided coverage of preventive dental services. None had prior experience with oral health education. Their overall comfort level with using computers and the Internet was low: *not comfortable at all* (25%), *not very comfortable* (41.7%), *quite comfortable* (25%), and *very comfortable* (8.3%).

### Quantitative findings post-intervention

The participants’ level of satisfaction with the educational intervention was high, with a mean of 18.8 (*SD* = 2.01; range, 15–20). After the intervention, all participants reported improved oral health knowledge and willingness to use preventive dental care. However, one-third of the sample stated that they could not find a dental clinic or make an appointment for preventive dental care on their own.

### Qualitative feedback post-intervention

Qualitative data offered rich information on participants’ experience with the intervention. Participant responses to the intervention yielded four themes: (1) content and structure, (2) linguistic and cultural aspects, (3) delivery mode, and (4) additional concerns and suggestions.

#### Content and structure

Participants expressed a high level of satisfaction with the intervention’s content, noting that it was informational, educational, and resourceful. A 76-year-old female participant (#1) stated, “The program was full of useful information. I learned a lot today. I particularly enjoyed learning how to use dental floss. I just realized that the way that I usually do it is not correct. The video kindly taught me how to floss properly. I didn’t know that I should move the floss around between the teeth like sawing.” Another participant (#4), who had been reluctant to use dental floss over the concern that it might create space between the teeth, exclaimed, “It was good to know that my concern was a common myth. Education made me feel much more comfortable with flossing.” A 77-year-old male participant (#2), who referred to himself as health conscious, said that the education confirmed his knowledge about oral health and dental care. Several participants commented positively on the website demonstrating how to find a dental clinic in their neighborhood. One participant (#3) stated, “The website was fascinating. I was shocked to know that there are over 90 Korean dental clinics in the 90,006 zip-code area, and all of these places are where I can use my Medi-Cal.” Participants expressed their satisfaction with the intervention’s general structure (e.g., its length, the narration’s voice and pace, the presentation’s graphics, and the use of audiovisual materials). The participants also recommended content coverage on dentures and implants (#12), interdental brushes (#7), and breath control (#10). Two participants (#3 and #8) suggested incorporating a Q&A session.

#### Linguistic and cultural aspects

All participants highly endorsed using Korean in the educational intervention. A 77-year-old female participant (#8) stated, “I immigrated to the United States more than 30 years ago, but I don’t speak English well. If the education was in English, I wouldn’t even have signed up for it.” Another participant (#6) stated, “Because it [the educational intervention] was in Korean, I could understand the content. The narration in Korean made me stay focused and fully engaged from the beginning to the end. If it was in English, I might have fallen asleep.” Calling attention to the particular challenge of understanding medical terminology in English, one participant (#7) stated, “Since it was in Korean, I didn’t have to worry about missing critical information or misunderstanding something.”

The participants also offered comments on cultural attitudes about preventive dental care. Reflecting a lack of understanding and low prioritization of preventive care, one participant (#2) said, “As the old saying says, you’d better stay away from doctors and clinics. I don’t usually see a doctor unless there is a critical need, such as bleeding and pain.” He also commented that he was uncomfortable with tasks in the U.S. healthcare system, such as making appointments and filling out forms. A female participant who was a lung cancer survivor (#4) provided a similar passive or avoidant response: “I was lucky to detect cancer at an early stage. That experience taught me how critical preventive care is. However, regarding dental care, my thoughts are mixed. What should I do if a major tooth problem is found during the check-up and its treatment is too expensive? This fear makes me wonder if it would be better not to know about the problem at all.” Alluding to the cultural belief that endurance is a virtue, one female participant (#3) shared her experience of losing a molar: “I had occasional pain in my molar but left it unattended for a long time. I just put up with the pain, thinking that my endurance would make it go away. When the pain reached the point that I could not bear it any longer, I finally saw a dentist, but it was too late. There was no other option than to extract the molar. I felt so bad that I let a small problem grow bigger.” She continued, “Teeth are an important part of your body, and having good teeth is a huge blessing. I blame myself for putting up with the pain instead of checking it with a dentist.”

#### Delivery mode

Despite varying comfort levels with using technology, all participants strongly endorsed the digital delivery of the education intervention. An 89-year-old female participant (#7) said, “The education through a computer was great. I simply followed the computer screen like I was watching TV, and it taught me lots of things. It was very easy to follow.” Participants commented positively on several intervention features: animation, narration, video clips, and linked information on the web. One participant (#1), who was fairly comfortable using technology, stated, “I really liked the computer-based education. It suits me well, but I am not sure if it would work with other seniors who do not know much about computers. Most of them would need hands-on assistance.” Indeed, several participants with low computer skills indicated that they could complete the education intervention only with the technical assistance offered by research personnel (e.g., equipment setup). When asked about other forms of advanced technology (e.g., mobile-app-based education), all but two participants (#1 and #4) expressed concerns about operating the technology independently. Several participants expressed their desire to retain a printed version of the educational materials in addition to the digital-based program. One participant (#2) said, “Although the world has changed tremendously, I still like reading things on paper. I would appreciate it if there was a small booklet that I could carry with me and refer to later on.” Another participant (#9) also said, “A booklet or handout would be useful when sharing the information with my friends.” There was strong support for individual learning; however, two participants (#4 and #9) spoke of the lack of opportunities for sharing experiences and exchanging opinions in a group.

#### Additional concerns and suggestions

After the oral health education intervention, although the quantitative assessment showed an overall improvement in oral health knowledge and willingness to use preventive care, several participants still referred to the difficulties in seeking preventive dental services. One theme that emerged was the need for navigational assistance. Participants with limited skills in English and computer use indicated a strong desire to have someone who could assist them navigating their dental service. One participant (#2) stated, “Even though the website shows a long list of dental clinics, you still have to call them to ensure that they accept a new patient with Medi-Cal. You have to check many things before you go, but I am not quite confident that I can do that all on my own.” Although his adult son was a major support, he remained concerned: “I don’t want to burden him. I usually try to do things on my own, but when it comes to seeing a doctor, I need his help. I wish there were some kinds of services that I could turn to.” One participant (#3), a new Medi-Cal enrollee, recalled, “When I applied for Medi-Cal, I received great help from a Korean social worker at the senior center. She took care of the complicated paperwork. I wish someone like her could offer assistance with finding the right clinic to visit.” A female participant (#11), who had no support besides her husband, also underscored the need for formal services to navigate dental care services.

A few participants were skeptical due to their adverse experiences in dental care settings. As one participant (#7) stated, “Several years ago, I went to a dental clinic with my Medi-Cal. I thought the service was free, but they charged me $160. They said it was for deep cleaning. It was an unexpected expense, and they hadn’t even asked me if I wanted that service.” Another participant (#9) said, “Maybe because I was a Medi-Cal patient and was no help for the clinic in making a profit, they treated me very poorly. I even noticed their rough hand movements during cleaning. It is very unpleasant!” She also spoke of a friend who received a derogatory comment from a dental assistant regarding her financial status. The participants also mentioned overtreatment (#8), denied or delayed service (#5, #9, and #11), and dissatisfaction with the service’s quality (#7). Unfair treatment, discrimination, and suboptimal care made older adults reluctant to use preventive care with Medi-Cal, even after our educational intervention.

## Discussion

The 12 older Korean Americans in our pilot sample generally faced English language barriers, with high oral health needs and varying levels of comfort with using technology. Over half of the sample was unaware of Medi-Cal’s preventive dental coverage, and none had prior exposure to oral health education. Participants were volunteers, 90% of whom had at least a high school education, with active social engagement (inferred from their attendance at a senior center). The post-intervention quantitative assessment yielded high participant satisfaction, with all reporting positive changes in their oral health knowledge and willingness to use preventive dental care. Their qualitative feedback contextualized these findings and provided insights for further intervention refinement.

Participants favorably evaluated the intervention’s content and structure, with consensus that its length, content, and audiovisual aids were appropriate. The YouTube video on how to use dental floss and the Medi-Cal dental provider directory website were also well received, helping participants apply their knowledge to action. All participants strongly endorsed using the Korean language, which enabled their active participation in the learning. This finding underscores the significance of language concordance in interventions with older immigrants with LEP [13, 14]. The use of an individual’s native language is particularly important in health education, given the challenges posed by medical terminology. Participants also noted the importance of incorporating cultural elements into the intervention. For example, beliefs about preventive care and pain management associated with the cultural values of endurance and acceptance [18–20] should be addressed in health interventions. To reinforce the benefits of preventive care, educational content must be enhanced by culturally reframing preventive health behaviors. Linguistic and cultural appropriateness is critical for individuals from racial and ethnic minority backgrounds [22].

Despite their varying levels of comfort with technology, all participants completed the educational intervention and strongly endorsed its digital delivery. Particularly high satisfaction was found among those with high levels of educational attainment and computer competency. During the pilot testing, research personnel provided technical assistance with setting up and operating the equipment. Four individuals with low computer competency were concerned that they could not have participated in the program had technical assistance been unavailable. Overall, our findings suggest a critical need for technical support and accommodation. Many participants requested a printed version (e.g., a booklet, pamphlet, or handout), but only two participants were interested in mobile-app-based education. Given the participants’ varying capabilities, needs, and desires, various types of educational delivery should be considered to effectively reach diverse groups within target populations.

The participants also mentioned factors that could potentially hinder preventive dental service use. First, many spoke of their limitation in navigating dental services. Due to language and information literacy barriers and other logistical challenges (e.g., unfamiliarity with U.S. healthcare systems), many older immigrants with LEP stated their need for additional support to navigate oral healthcare. Participants also expressed their need for formal services to reduce their burden on their families. Thus, improved knowledge by itself might be insufficient for positive changes in individuals’ actions, suggesting the need for a navigation assistance program (e.g., support in finding dental clinics that accept Medi-Cal, making appointments, communicating with healthcare professionals).

Adverse experiences using Medi-Cal were another critical barrier that emerged in the participants’ interviews. A few reported direct or indirect unfair treatment, discrimination, and suboptimal care when using Medi-Cal’s medical services, discouraging them from seeking other healthcare services to which they were entitled. According to the aforementioned CHIS-based study [16], more than 36% of adult Medi-Cal beneficiaries experienced discrimination in healthcare settings, reducing the odds of using dental care services by 18%. Discriminatory experiences as a barrier to healthcare services are common in socially disadvantaged groups [25–27]. These findings call for cultural and systematic changes in patient–provider interactions and public support mechanisms. Without such changes, it is difficult to expect that oral health education interventions to translate the improved knowledge into behavioral outcomes (i.e., preventive care use).

Several limitations of this study should be noted. First, it focuses on a single ethnic group with a small sample from one geographic location. Although the findings are not generalizable to a larger population, the study provides enriched data to contextualize the target group’s unique characteristics and needs. Second, as a community-based volunteer sample, participants tended to be highly educated, physically and cognitively intact, and socially engaged. In addition, social desirability in responses should be considered when interpreting the findings.

## Conclusion

In response to the lack of knowledge about oral health and insurance coverage resulting in underutilizing dental benefits among older immigrants, we developed a digital oral health intervention for older Korean-American Medi-Cal enrollees in Los Angeles. This study demonstrates developing and refining an oral health education program to promote preventive dental services among publicly insured yet underserved populations. Based on this pilot test, the intervention’s technical, linguistic, and cultural appropriateness will be enhanced. Future efforts will be directed toward the intervention’s efficacy and effectiveness and comparing outcomes using different delivery modes.

## Data Availability

The dataset used in the current study is available from the corresponding author upon request.
